# Deficiencies in both starch synthase IIIa and branching enzyme IIb lead to a significant increase in amylose in SSIIa-inactive japonica rice seeds

**DOI:** 10.1093/jxb/eru310

**Published:** 2014-07-28

**Authors:** Hiroki Asai, Natsuko Abe, Ryo Matsushima, Naoko Crofts, Naoko F. Oitome, Yasunori Nakamura, Naoko Fujita

**Affiliations:** ^1^Department of Biological Production, Akita Prefectural University, Akita City, Akita, 010-0195, Japan; ^2^Institute of Plant Science and Resources, Okayama University, Kurashiki, Okayama, 710-0046, Japan

**Keywords:** Amylopectin, amylose, branching enzyme, mutant, rice, starch synthase.

## Abstract

Starch synthase (SS) IIIa has the second highest activity of the total soluble SS activity in developing rice endosperm. Branching enzyme (BE) IIb is the major BE isozyme, and is strongly expressed in developing rice endosperm. A mutant (*ss3a/be2b*) was generated from wild-type japonica rice which lacks SSIIa activity. The seed weight of *ss3a/be2b* was 74–94% of that of the wild type, whereas the *be2b* seed weight was 59–73% of that of the wild type. There were significantly fewer amylopectin short chains [degree of polymerization (DP) ≤13] in *ss3a/be2b* compared with the wild type. In contrast, the amount of long chains (DP ≥25) connecting clusters of amylopectin in *ss3a/be2b* was higher than in the wild type and lower than in *be2b*. The apparent amylose content of *ss3a/be2b* was 45%, which was >1.5 times greater than that of either *ss3a* or *be2b*. Both SSIIIa and BEIIb deficiencies led to higher activity of ADP-glucose pyrophosphorylase (AGPase) and granule-bound starch synthase I (GBSSI), which partly explains the high amylose content in the *ss3a/be2b* endosperm. The percentage apparent amylose content of *ss3a* and *ss3a/be2b* at 10 days after flowering (DAF) was higher than that of the wild type and *be2b*. At 20 DAF, amylopectin biosynthesis in *be2b* and *ss3a/be2b* was not observed, whereas amylose biosynthesis in these lines was accelerated at 30 DAF. These data suggest that the high amylose content in the *ss3a/be2b* mutant results from higher amylose biosynthesis at two stages, up to 20 DAF and from 30 DAF to maturity.

## Summary

Deficiencies in both starch synthase IIIa and branching enzyme IIb lead to significantly increased amylose content in japonica rice endoseprm due to pleiotropic effects on other starch biosynthetic enzymes.

## Introduction

Accumulated starch in plant storage tissues is widely used for food manufacturing and industrial applications. Starches contain glucose homopolymers of primarily linear amylose chains and branched amylopectin chains. The amylose content greatly affects the physicochemical properties of starch (Singh *et al.*, 2006), and starches with different amylose contents are used for different functions. For example, resistant starch (RS) contains high levels of amylose and long-chain amylopectin, is resistant to the hydrolase used for low-calorie food manufacturing, and is used in the prevention of colon cancer and diabetes (Bird *et al.*, 2004; Regina *et al.*, 2006).

At least four enzymes participate in starch biosynthesis, namely ADP-glucose pyrophosphorylase (AGPase), starch synthase (SS), branching enzyme (BE), and debranching enzyme (DBE) (Smith *et al.*, 1997; Nakamura, 2002). These four enzymes have multiple isoforms. Of these enzymes, granule-bound starch synthase I (GBSSI) is involved in amylose biosynthesis, and the remaining enzymes are involved in amylopectin biosynthesis. BE is the only enzyme that forms branch points in amylopectin molecules. SSI, SSIIa, and SSIIIa are required for the synthesis of amylopectin in maize and rice; they elongate amylopectin chains with degree of polymerization (DP) 6–7 to DP 8–12, DP 6–12 to DP 13–24, and long chains connecting amylopectin clusters, respectively (Nakamura *et al.*, 2005; Fujita *et al*., 2006, 2007). Of these SS isozymes, SSIIa is inactive in japonica cultivars (Nakamura *et al.*, 2005).

These reports suggest that SSs except for GBSSI and BEs have central roles in amylopectin biosynthesis. Recently, mutant rice lines were isolated by crossing mutants in SSI and BEI or BEIIb, and the starch structure (Abe *et al.*, 2014) and physicochemical properties (Abe *et al.*, 2013) of the mutant were analysed. Although the complete deficiency of SSI and BEIIb led to sterility, the recessive mutant (*ss1* ^*L*^ */be2b*; leaky *ss1* mutation and null *be2b* mutation) was fertile, and its seeds had a greater weight than those of the *be2b* mutant (Abe *et al.*, 2014). The increased amylose content in *be2b* was due to reduced amylopectin biosynthesis. However, reduction of SSI activity in the BEIIb deficiency background might lead to correction of the branching and elongation imbalance found in the mutant, which would ultimately enhance amylopectin biosynthesis. Deficiency of SSI and/or BEI resulted in minor effects on seed weight, starch accumulation, and amylose content. Analysis of the amylopectin chain-length distribution of *ss1/be1* endosperm showed that the effects of SSI and BEI on amylopectin structure are additive (Abe *et al.*, 2014). These results indicate that different amylopectin structures are produced depending on the combinations of active SS and BE isozymes.

In the present study, the mutant line (*ss3a/be2b*) was generated in the SSIIa-inactive japonica background by crossing mutant lines in SSIIIa, which has the second highest SS activity in the soluble fraction from developing endosperm of rice, and BEIIb, which has a distinct role in formation of the branch point in the crystalline lamellae of amylopectin. Surprisingly, the amylose content of the *ss3a/be2b* mutant was significantly higher compared with that of the mutant parental lines. The synthesis of amylose and amylopectin during endosperm development is discussed in light of these results.

## Materials and methods

### Plant materials

**Fig. 1.**
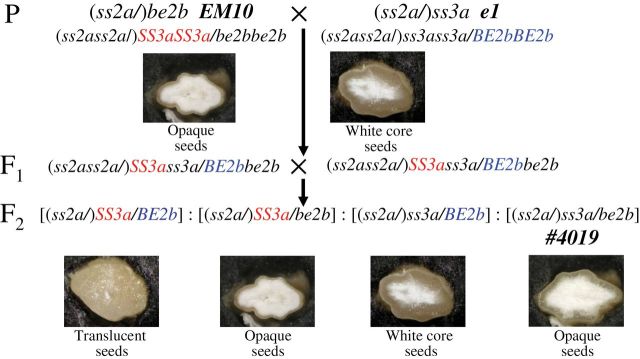
Pedigree and seed morphologies of the mutant lines. The morphology of rice seed cross-sections of the *ss3a/be2b* mutant line and the parental mutant lines (*be2b* and *ss3a*). The genotypes of the *SSIIa* gene are shown in parentheses.

To generate the *ss3a/be2b* mutant line, the mutant japonica lines *ss3a* [SSIIIa-deficient mutant (*e1*); Fujita *et al.*, 2007] and *be2b* [BEIIb-deficient japonica rice mutant (*EM10*); Nishi *et al.*, 2001] were used in crosses. The cultivars Nipponbare (the parent of *e1*) and Kinmaze (the parent of *EM10*) were used as controls. The resulting double heterozygotes (F_1_) were self-pollinated (Fig. 1). Recessive mutant F_2_ seeds (*ss3a/be2b*) were selected on the basis of their opaque-seed phenotype, and confirmed by performing native-polyacrylamide gel electrophoresis (PAGE)/SS-activity staining of developing endosperm and by immunoblot analysis of mature endosperm. Rice plants were grown during the summer in an experimental paddy field at Akita Prefectural University under natural environmental conditions.

### Native-PAGE/SS-activity staining, enzyme assays, and immunoblotting

Native-PAGE/SS-activity staining of BE and DBE was performed as described previously (Yamanouchi and Nakamura, 1992; Fujita *et al.*, 1999). SS-activity staining was performed on 7.5% (w/v) acrylamide slab gels containing 0.8% (w/v) oyster glycogen (G8751, Sigma) according to the protocol of Nishi *et al.* (2001), with the additional inclusion of 0.5M citrate. Assays for AGPase and GBSSI of developing endosperm [10 days after flowering (DAF)] were performed as described by Nakamura *et al.* (1989) and Fujita *et al.* (2001).

Immunoblotting was performed as previously described by Crofts *et al.* (2012) using antiserum raised against SSI (Fujita *et al.*, 2006), SSIIIa (Crofts *et al.*, 2012), GBSSI (Fujita *et al.*, 2006), BEI (Satoh *et al.*, 2003), and BEIIb (Nishi *et al.*, 2001).

### Protein extraction from developing and mature endosperm

Total proteins were extracted from developing endosperm from three individuals (12 DAF) per line using 200 μl of urea buffer [125mM TRIS-HCl (pH 6.8), 8M urea, 4% (w/v) SDS, and 5% (v/v) 2-mercaptoethanol] and a plastic pestle. The homogenate was incubated with a rotator (Iuchi MTR-103, Japan) at 37 °C for 2h. The homogenate was centrifuged at 20 000 *g* at room temperature for 20min and the supernatant was set aside. The pellet was homogenized in 200 μl of urea buffer, and then centrifuged under the same conditions. The pooled supernatants were loaded on SDS–PAGE and the proteins were stained with Coomassie brilliant blue (CBB). The amounts of total proteins were normalized to the same intensity of general protein bands on the SDS–polyacrylamide gel stained with CBB, and used for immunoblotting.

Soluble protein (SP) and loosely bound protein (LBP) fractions were prepared from developing (12 DAF) and mature endosperm as follows: seeds were ground in 3 vols of extraction buffer [50mM imidazole-HCl (pH 7.4), 8mM MgCl_2_, 50mM 2-mercaptoethanol, and 12.5% (v/v) glycerol] to obtain the supernatant. The resulting pellet after centrifugation at 20 000 *g* for 10min at 4 ºC was extracted with 2 vols of the same buffer twice. These supernatants of the three extractions were combined and defined as the SP fraction. The resulting pellet after extractions of the SP fraction was extracted with 3 vols of SDS solution [55mM TRIS-HCl (pH 6.8), 10% SDS, 5% 2-mercaptoethanol, and 12.5% (v/v) glycerol] and the resulting pellet after centrifugation at 20 000 *g* for 10min at 4 ºC was extracted with 2 vols of SDS solution twice. These supernatants of the three extractions were combined and defined as the LBP fraction. The tightly bound protein (TBP) fraction was extracted from the 3mg of pellet after extraction of the LBP fraction by boiling with 30 μl of SDS solution for 7min. The resulting gel was extracted with 60 μl of SDS solution and the suspension was centrifuged at 20 000 *g* for 10min at 4 ºC to obtain the TBP as described in Fujita *et al.* (2006).

### Analysis of starch and amylopectin structure

Starch was extracted from mature rice endosperm to assess the amylopectin chain-length distribution according to the method of Fujita *et al.* (2001). The chain-length distribution of endosperm starch was analysed by capillary electrophoresis (O’Shea and Morell, 1996) using the P/ACE MDQ Carbohydrate System (Beckman Coulters, CA, USA).

Gel filtration chromatography of starches (from mature and developing endosperm) and amylopectin (from mature endosperm) was performed as described previously (Fujita *et al.*, 2007, 2009) using a Toyopearl HW55S gel filtration column (300×20mm) connected in series to three Toyopearl HW50S columns (300×20mm) equipped with a refractive index (RI) detector (Tosoh RI-8020).

Estimation of starch content in rice seeds and determination of the amylopectin molecular weight were performed by HPSEC-MALLS-RI according to the method of Fujita *et al.* (2003). Purified starch granules were coated with gold using a fine coater (JEOL JFC-1200) for 120 s. Starch granule morphology was examined by scanning electron microscopy (SEM; JEOL-500, Tokyo, Japan). SEM was performed in a secondary electron mode at 15kV according to the method of Fujita *et al.* (2003). Observation of iodine-stained endosperm thin sections was performed according to the method of Matsushima *et al.* (2010).

## Results

### Generation of recessive mutant lines (*ss3a*/*be2b*)

**Fig. 2.**
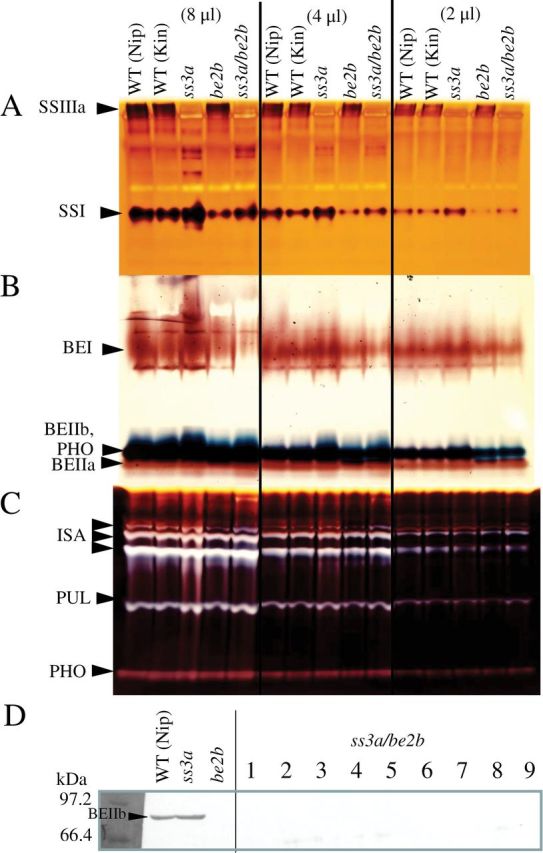
Enzyme activities on zymograms and immunoblotting. Native-PAGE/SS-activity staining of starch synthases (SSs; A), branching enzymes (BEs; B), and debranching enzymes (DBEs; C), and immunoblotting using anti-BEIIb serum (D), of developing endosperm at 12 days after flowering from the *ss3a/be2b* mutant, parental mutant, and wild-type lines. SSI, SSIIIa, BEI, BEIIa, BEIIb, ISA (isoamylase), PUL (pullulanase), and PHO (phosphorylase) activity bands are indicated by arrowheads. Crude extracts were prepared by grinding the developing endosperm with 3 vols of extraction solution per milligram fresh weight. The volumes of crude extract applied to the native gels were 8, 4, and 2 μl as described at the top of the figure. Kin, Kinmaze; Nip, Nipponbare; WT, wild type.

To generate a recessive mutant line (*ss3a/be2b*, *#4019*), the *be2b*-null mutant (*EM10*; Nishi *et al.*, 2001) was crossed with the *ss3a*-null mutant (*e1*; Fujita *et al.*, 2007; Fig. 1). The selected F_2_ seeds (*ss3a/be2b*) were grown and self-pollinated. The developing endosperm from F_3_ seeds lacked the SSIIIa activity band on native-PAGE/SS activity staining gels (Fig. 2A), and it also lacked the BEIIb band measured by immunoblotting assays using antiserum against BEIIb (Fig. 2D; Nishi *et al.*, 2001). F_3_ and F_4_ seeds from recessive mutant lines were used for further analyses. Since all rice lines used in this study have inactive SSIIa derived from japonica rice cultivars, the notation of the genotype of the *SSIIa* gene was omitted throughout the text, figures, and tables, except for Fig. 1.

### Pleiotropic effects of SSIIIa and BEIIb deficiencies on other starch biosynthetic enzymes

**Fig. 3.**
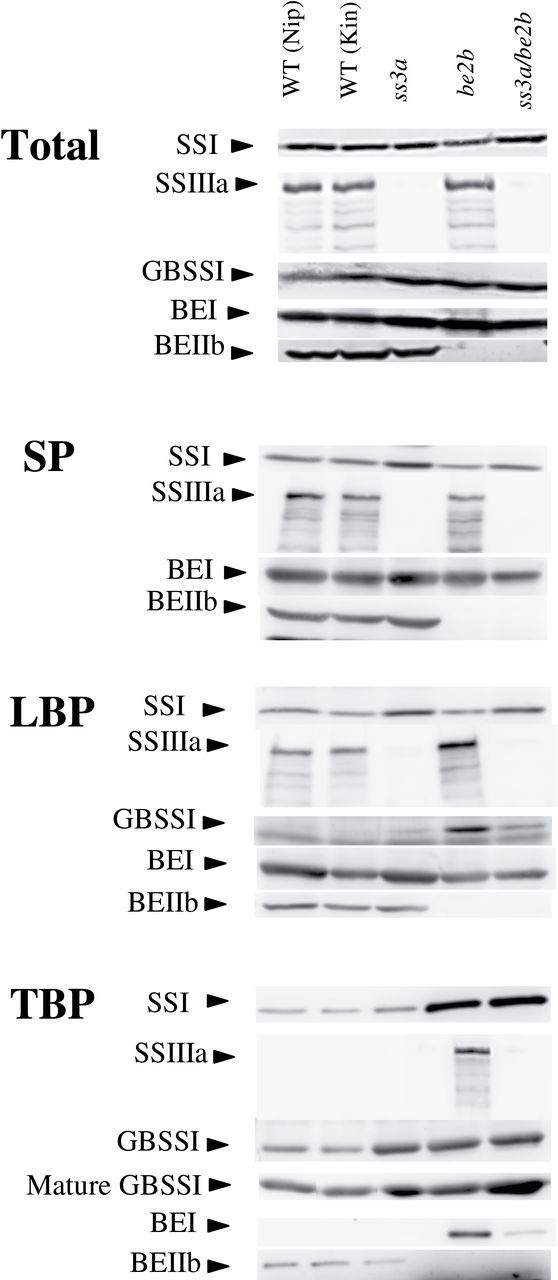
Isozyme distributions in protein fractions from developing endosperm. Immunoblotting of the total protein extract (Total), the soluble protein fraction (SP), the loosely bound protein fraction (LBP), and the tightly bound protein fraction (TBP) from developing endosperm at 12 days after flowering (DAF) of the *ss3a/be2b* mutant, parental mutant, and wild-type lines using antiserum raised against rice SSI, SSIIIa, GBSSI, BEI, and BEIIb. GBSSI from mature endosperm (mature GBSSI) was also analysed. The amount of protein in the Total, SP, and LBP bands was standardized by one seed, and TBP was standardized by milligrams of starch. Kin, Kinmaze; Nip, Nipponbare; WT, wild type.

To test the effects of SSIIIa and/or BEIIb deficiency on other starch biosynthesis isozymes, semi-quantitative native-PAGE/SS-activity staining gel analysis (Fig. 2) and immunoblotting assays were performed on the soluble protein fraction (Fig. 3, SP) from developing endosperm 12 DAF. SSI activity was ~1.5 times higher in *ss3a* compared with the wild type (Fig. 2A; Fujita *et al.*, 2007), whereas SSI activity was ~50% lower in *be2b* compared with the wild type (Fig. 2A; Nishi *et al.*, 2001; Abe *et al.*, 2014). The SSI protein level in the SP fraction (Fig. 3) was also related to the SSI activity level (Fig. 2). A reduction in SSI activity and total SS activity was demonstrated previously in the rice *be2b* (*EM10*) mutant (Nishi *et al.*, 2001) and in maize (Boyer and Preiss, 1978). The SSI activity level in the *ss3a/be2b* mutant was slightly lower than that of the wild-type cultivars (Fig. 2A). The BEI activity bands of *ss3a* were slightly higher than that of the wild-type cultivar, whereas the BEI activity bands of *be2b* and *ss3a/be2b* were slightly lower than those of the wild-type cultivars (Fig. 2B). The activities of the other starch biosynthesis isozymes [BEIIa (Fig. 2B), ISA, PUL, and PHO1 (Fig. 2C)] were not significantly different between the mutant lines and the wild-type cultivars.

The pleiotropic effects of SSIIIa and/or BEIIb deficiency on the activities of AGPase (which produces ADP-glucose) and GBSSI (amylose synthase) in developing endosperm (10 DAF) were examined (Table 1). AGPase activities of parental mutant lines were ~1.5 times higher than that of the wild-type cultivar. The AGPase activity of *ss3a/be2b* was more than twice higher than that of the wild-type cultivar. GBSSI activity per milligram of starch of *ss3a* and *ss3a/be2b* was ~2-fold higher than that of the other lines, although that of *be2b* was comparable with the wild type (Table 1).

**Table 1.** Enzyme activities of AGPase and GBSSI of developing endosperm (10 DAF) and the amount of GBSSI protein of mature endosperm estimated by immunoblotting bands of the rice *ss3a/be2b* mutant line, the parental mutant *ss3a* and *be2b* mutant line, and the wild-type (WT) rice Nipponbare and Kinmaze

Lines	AGPase^*a*^ (nmol min^–1^ endosperm^–1^)	GBSSI^*a*^ (nmol min^–1^ mg starch^–1^)	GBSSI protein amount^*d*^ (relative values)
WT (Nipponbare)	0.24±0.01^*f*^ (100)^*b*^	0.15±0.03^*f*^ (100)^*b*^	100^*b*^
WT (Kinmaze)	0.20±0.04^*f*^ (100)^*c*^	0.22±0.01 (100)^*c*^	100^*c*^
*ss3a*	0.37±0.02^*e*^ (154)^*b*^	0.29±0.03^*e*^ (193)^*b*^	129.7^*b*^
*be2b*	0.31±0.07 (155)^*c*^	0.21±0.03 (95)^*c*^	167.7^*c*^
*ss3a/be2b*	0.51±0.08 (213)^*b*^	0.27±0.02 (180)^*b*^	291.8^*b*^

^*a*^ Mean± SE of three seeds.

^*b*^ Percentage of the wild type (Nipponbare).

^*c*^ Percentage of the wild type (Kinmaze).

^*d*^ The GBSSI protein amount of immunoblotting bands from the TBS fraction of mature seeds (Fig. 3) was quantified by MultiGauge ver. 3.0 software (Fuji film, Japan).

^*e*^ Significant differences between parental mutant lines and the wild type by *t*-test at *P*<0.05.

^*f*^ Significant differences between the *ss3a/be2b* mutant line and the WT by *t*-test at *P*<0.05.

To determine the relative abundance of isozymes involved in starch biosynthesis in rice endosperm, including SSI, BEI, BEIIb, SSIIIa, and GBSSI, total proteins were extracted with urea buffer from different mutant lines and were used for immunoblotting (Fig. 3, Total). The results show that the levels of GBSSI protein in *ss3a*, *be2b*, and *ss3a/be2b* mutants are slightly higher than those in the corresponding wild-type cultivars. For all other isozymes tested, the protein levels in the mutants were not significantly different from those in the corresponding wild-type cultivars.

Next, the pleiotropic effects that BEIIb and/or SSIIIa deficiencies have on protein levels in LBP and TBP fractions from developing endosperm were examined. The levels of SSI, SSIIIa, GBSSI, BEI, and BEIIb in the LBP fraction and the TBP fraction were determined by immunoblotting (Fig. 3, LBP and TBP). The amount of SSI in the LBP fraction was higher in *ss3a* and *ss3a/be2b* than in the wild type (Fig. 3, LBP). The amounts of SSIIIa and GBSSI in the LBP fraction were higher in *be2b* than in the wild type. In the TBP fraction, SSI was significantly higher in BEIIb-deficient mutant lines (*be2b* and *ss3a/be2b*) than in the other lines or the wild type (Fig. 3, TBP). SSIIIa was detected in the TBP fraction in *be2b*, but not in the wild type (Fig. 3, TBP). The BEI bands detected in the TBP fractions from *be2b* and *ss3a/be2b* were dense and faint, respectively. GBSSI levels in the TBP fractions of immature and mature seeds were higher in *ss3a*, *be2b*, and *ss3a/be2b* than in the wild type (Fig. 3, TBP). The GBSSI protein amount in mature seed from *ss3a/be2b* (Fig. 3) was significantly higher (291.8% of the wild type) (Table 1).

### Grain weight, starch content, and molecular weight of amylopectin

Of the three BE isozymes expressed in rice endosperm, BEI and BEIIb are the major isozymes for branching of amylopectin molecules. A deficiency in BEIIb induces a more pronounced phenotype than deficiencies in either BEI or BEIIa (Nishi *et al.*, 2001; Nakamura, 2002; Satoh *et al.*, 2003). The seed weight is known to depend on environmental conditions and genetic background. Therefore, the dehulled grain weights of seeds from the mutant *ss3a/be2b*, the parental mutants, and the wild-type lines were measured for 3 years (2008–2010; Table 2). The seed starch content was also measured. Dehulled grain weight and starch content were greatly reduced in *be2b* mutant seeds, and were 59.3–72.6% and 49.2% of the levels in the wild type, respectively (Table 2). The dehulled grain weight and starch content in *ss3a* mutant seeds were 102.6–102.9% and 95.2% of the levels of wild type, respectively (Table 2). These results were consistent with previous reports (Nishi *et al.*, 2001; Fujita *et al.*, 2007; Abe *et al.*, 2014). Surprisingly, in the *ss3a/be2b* mutant, dehulled grain weight and starch content were greater than those in the *be2b* mutant, and were 74.4–93.7% and 78.6% of the levels in the wild type, respectively (Table 2).

**Table 2.** Duhulled grain weight, starch content, amylose content, weight average molecular weight, and z-average radius of gyration of amylopectin in the rice *ss3a/be2b* mutant line, the parental mutant lines, and the wild-type (WT) lines

Lines	Grain weight^*a*^ (mg)			Starch content^*b*^ (mg)	M_w_^*c*^ (×10^8^ g)	R_z_^*d*^ (nm)	Amylose content^*e*^ (mg per seed)
	2008	2009	2010	2010			
WT (Nipponbare)	19.7±0.2^*j*^ (100.0)^f^	19.0±0.2^*j*^ (100.0)^*f*^	21.1±0.2^*j*^ (100.0)^*f*^	12.6±0.7^*j*^ (100.0)^*f*^	43.7±4.1^*j*^ (100.0)^*f*^	665.4±26.9^*j*^	2.7
WT (Kinmaze)	19.4±0.2^*j*^ (100.0)^*g*^	19.8±0.2^*j*^ (100.0)^*g*^	20.2±0.2^*j*^ (100.0)^*g*^	13.0±0.3 (100.0)^*g*^	–	–	2.8
*ss3a*	20.3±0.2^*h*,*i*^ (102.9)^*f*^	19.5±0.2^*i*^ (102.6)^*f*^	20.5±0.2^*h*,*i*^ (102.9)^*f*^	12.0±0.4 (95.2)^*f*^	10.4±0.5^*h*,*i*^ (23.8)^*f*^	451.6±8.9^*h*,*i*^	3.7
*be2b*	11.7±0.3^*h*,*i*^ (59.3)^*g*^	13.8±0.2^*h*,*i*^ (72.6)^*g*^	13.5±0.2^*h*,*i*^ (64.0)^*g*^	6.4±0.1^*h*^ (49.2)^*g*^	5.3±1.2^*h*^ (12.1)^*f*^	417.3±23.5^*h*,*i*^	1.8
*ss3a/be2b*	16.3±0.1 (82.7)^*f*^	17.8±0.2 (93.7)^*f*^	15.7±0.1 (74.4)^*f*^	9.9±0.9 (78.6)^*f*^	2.7±0.4 (6.2)^*f*^	326.5±15.8	4.5

^*a*^ Mean ±SE of 50 seeds.

^*b*^ Mean ±SE of three seeds.

^*c*^ Weight-average molecular weight (mean±SE of three replicates).

^*d*^ *z*-average radius of gyration (mean±SE of three replicates).

^*e*^ Amylose content per seed=starch content^*b*^×apparent amylose content (%) (Supplementary Table S1 at *JXB* online).

^*f*^ Percentage of the wild type (Nipponbare)

^*g*^ Percentage of the wild type (Kinmaze)

^*h*^ Significant differences between parental mutant lines and the wild type by *t*-test at *P*<0.05.

^*i*^ Significant differences between the parental mutant lines and the *ss3a/be2b* mutant by *t*-test at *P*<0.05.

^*j*^ Significant differences between the *ss3a/be2b* mutant line and the wild type by *t*-test at *P*<0.05.

The molecular weight of *ss3a* amylopectin was only 23.8% of that of the wild type (Table 2), in line with a previous report (Fujita *et al.*, 2007). The amylopectin molecular weight was 12.1% of that of the wild type in *be2b* but only 6.2% in *ss3a/be2b* (Table 2).

### Characterization of starch structure in mature endosperm of mutant lines

**Fig. 4.**
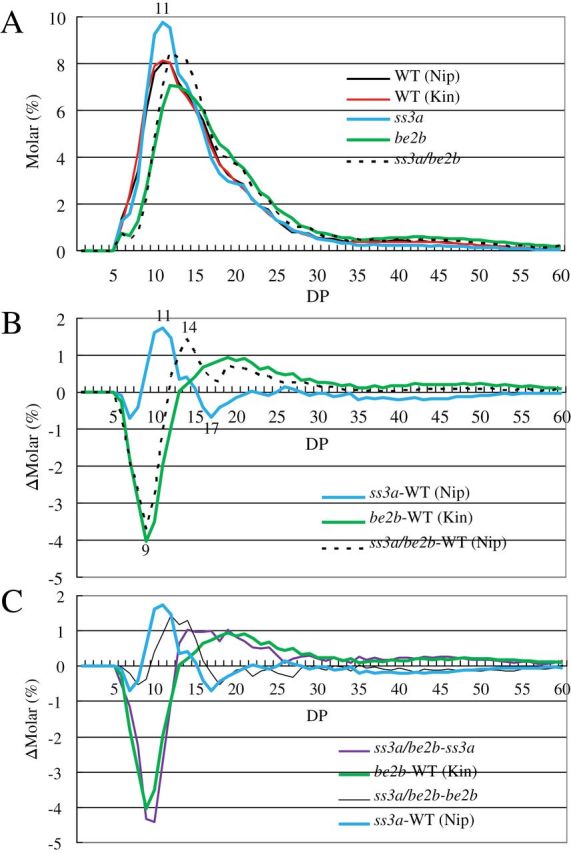
Analysis of amylopectin molecular structure by capillary electrophoresis. (A) Chain-length distribution patterns of amylopectin in mature endosperm. (B) Differential plots between mutant and wild-type lines. (C) Differential plots between the *ss3a/be2b* mutant and parental mutant lines. The numbers on the plots represent the DP values. Each figure shows one representative data set (one of at least three replicates using varied preparations from different rice seeds of homogenous plants). The relative standard error of molar % of each chain length in the range of DP5–60 was <5.7%. DP, degree of polymerization; Kin, Kinmaze; Nip, Nipponbare; WT, wild type.

To compare the structure of amylopectin among the *ss3a/be2b* mutant line, their parental mutant lines, and the wild-type lines, the amylopectin chain-length distribution was determined in endosperm using capillary electrophoresis (Fig. 4). In the range of DP ≤24 (within one cluster of amylopectin), the chain-length distribution pattern in *ss3a/be2b* was similar, but not identical to, that in *be2b*. Short chains with DP ≤12 were significantly less abundant in *ss3a/be2b* compared with the wild type (Fig. 4A, B). The amount of chains with DP 9–15 was much higher in *ss3a/be2b*, whereas the levels of DP 17 were lower, compared with the levels in *be2b* due to the SSIIIa deficiency (Fig. 4B). In the range of DP >24 (chains connecting more than two clusters of amylopectin), the amount of these long chains in *ss3a* and *be2b* mutant lines was significantly lower and higher, respectively, compared with that of the wild type (Fig. 4B; Nishi *et al.*, 2001; Fujita *et al.*, 2007). Conversely, the amount of chains with DP >24 in *ss3a/be2b* was intermediate with respect to the amounts in both parental mutant lines (Fig. 4B). The differential plot obtained by subtraction of the chain-length distribution of *ss3a* from that of *ss3a/be2b* was nearly identical to the corresponding differential plots of *be2b* versus the wild type (Fig. 4C). This indicates an additive effect of loss of SSIIIa activity on the amylopectin chain-length distribution in the *be2b* background. In contrast, the differential plot obtained by subtraction of the chain-length distribution of *be2b* from that of *ss3a/be2b* was shifted to the right compared with that of *ss3a* versus the wild type (Fig. 4C). The right-hand shift in the amylopectin chain-length distribution with DP ≤24 in *ss3a/be2b* with subtraction of *ss3a* implies that SSI elongates longer chains in the *be2b* background than in the wild type.

**Fig. 5.**
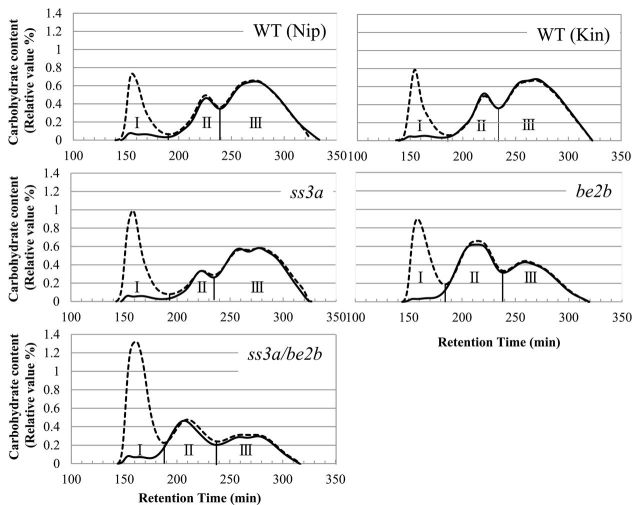
Size separation of debranched endosperm starch and purified amylopectin. Gel filtration chromatography was performed on debranched endosperm starch and purified amylopectin from the *ss3a/be2b* mutant, parental mutant, and wild-type lines. Each graph shows the typical elution profiles of isoamylase-debranched starch (dotted lines) and purified amylopectin (solid lines). Each fraction (Fr. I, II, and III) is separated according to the carbohydrate content curve determined by refractive index detectors (left *y*-axis). The panels show one typical data set (one of at least three replicates prepared from purified starches and amylopectin). Kin, Kinmaze; Nip, Nipponbare; WT, wild type.

To investigate further the components of starch and its structure in the mutant lines, the isoamylolysates of endosperm starch and purified amylopectin were subjected to size-exclusion chromatography using Toyopearl HW55S and HW50S (Fig. 5). In this study, the method of size-exclusion chromatography of debranched starch was used for the estimation of amylose content, because the conventional blue value method would overestimate the amylose content due to the long branched structures present in *ae* amylopectin (Nishi *et al.*, 2001). The λ_max_ values of the α-glucan–iodine complex >600nm indicated that fraction I (Fr. I) contained most, if not all, of the amylose (data not shown). A small amount of carbohydrate also was detected in Fr. I of the purified amylopectin sample (Fig. 5) and was designated as extra-long chain (ELC) amylopectin with DP ≥500 (Takeda *et al.*, 1987; Horibata *et al.*, 2004). Therefore, Fr. I from endosperm starch contains both true amylose and ELC amylopectin. The value obtained by subtraction of the ELC content from the apparent amylose content (AAC) of starch is equivalent to the true amylose content of starch (Horibata *et al.*, 2004). Fr. II contained B_2_–_3_ long chains of amylopectin connecting tandem clusters of amylopectin, whereas Fr. III contained short chains within one cluster of amylopectin. The proportion of each starch component was calculated based on the data shown in Fig. 5, and the results are presented in Supplementary Table S1 available at *JXB* online.

AAC values for *ss3a* (30.7%) and *be2b* (28.1%) were significantly higher than those in the wild-type lines Nipponbare (21.2%) and Kinmaze (21.6%) (Fig. 5; Supplementary Table S1 at *JXB* online), consistent with previous reports (Yano *et al.*, 1985; Fujita *et al.*, 2007). Surprisingly, the AAC value of *ss3a/be2b* (45.1%) was much higher than that of the parental mutant lines, and more than twice higher than the level in the wild type (Fig. 5; Supplementary Table S1). However, the amount of ELC in *ss3a/be2b* (2.5%) was similar to that of the wild type (3.0% in Nipponbare and 2.3% in Kinmaze). The ratios of Fr. III to Fr. II (III/II) in endosperm amylopectin of *ss3a* (4.4) and *be2b* (0.8) parental mutant lines were significantly higher and lower, respectively, than those in the wild type (Supplementary Table S1). The III/II ratio also was significantly lower in *ss3a/be2b* (0.9) due to the significantly decreased amylopectin short chains resulting from BEIIb deficiency.

### The morphology of starch granules

Purified starch granules from various mutant lines were observed by SEM (upper panels in Supplementary Fig. S1 at *JXB* online). Thin sections of seeds stained with iodine were also observed by light microscopy (lower panels in Supplementary Fig. S1). Several polygonal starch granules were packed in an amyloplast in the wild type and *ss3a*, although some starch granules were spherical in the *ss3a* mutant line (Fujita *et al.*, 2007). In contrast, some starch granules in *be2b* seeds were much larger than those in the wild type (upper panels in Supplementary Fig. S1; Abe *et al.*, 2013). From observations of the thin sections stained with iodine (lower panels in Supplementary Fig. S1), these starch granules seem to be multiple starch granules that have aggregated. Some starch granules in *ss3a/be2b* were peanut-shaped; such granules are also observed in high-amylose maize (Jiang *et al.*, 2010*b*). These starch granules also appeared to be aggregates of multiple starch granules (lower panels in Supplementary Fig. S1). The starch granule surface in *ss3a/be2b* was smooth, whereas that in *be2b* was rough (upper panels in Supplementary Fig. S1).

### Changes in seed weight, amylose content, and amylopectin content during seed development

**Fig. 6.**
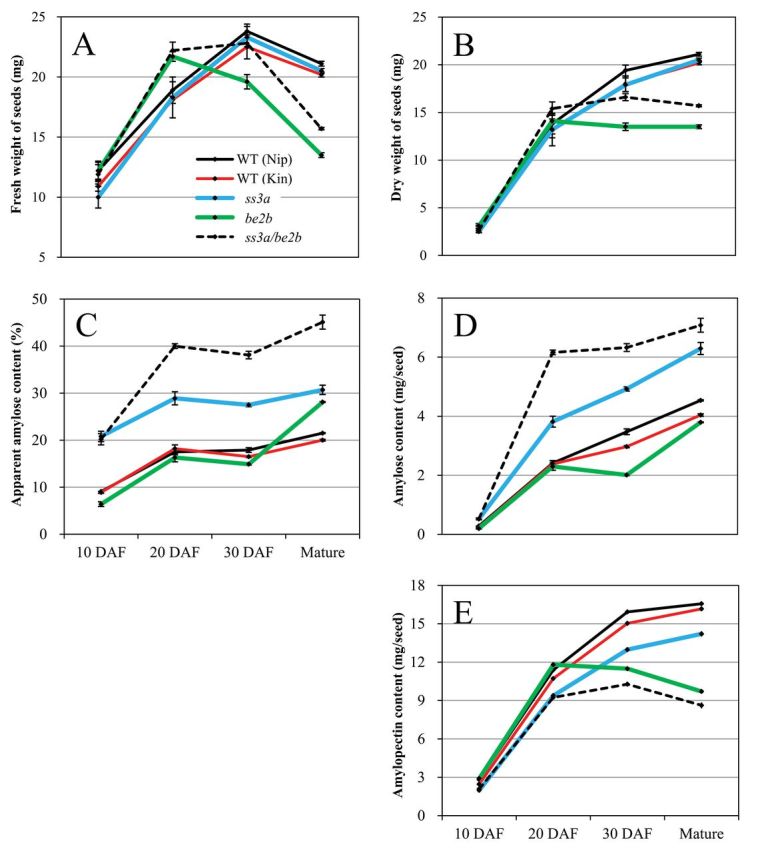
Changes in seed fresh weight, dry weight, amylose content, and amylopectin content during seed development. Amylose and amylopectin content per seed were calculated from the amylose and amylopectin content (%) and dry weight of seed at various stages of development. Error bars are the standard errors of three replications. Kin, Kinmaze; Nip, Nipponbare; WT, wild type; DAF, days after flowering.

The changes in seed fresh weight, dry weight, amylose content, and amylopectin content during seed development were examined at 10, 20, and 30 DAF, and in mature seeds (Fig. 6). The fresh weight of the wild type and *ss3a* mutant increased up to 30 DAF; after that, seed dehydration started (Fig. 6A). Seed dehydration started earlier (after 20 DAF) in BEIIb-deficient mutant lines (*be2b* and *ss3a/be2b*). The dry weight of BEIIb-deficient mutant lines did not increase after 20 DAF, whereas the dry weights of other lines continued to increase until maturity. The dry weight of *ss3a/be2b* seeds was constantly greater than that of *be2b* seeds during development (Fig. 6B).

The AAC (%) of SSIIIa-deficient lines (*ss3a* and *ss3a/be2b*) was already high (>20%) at 10 DAF, whereas that of the other lines was still low (<10%) at 10 DAF (Fig. 6C). The AAC (%) increased rapidly from 10 DAF to 20 DAF in every line, and that of BEIIb-deficient mutant lines also increased rapidly from 30 DAF to maturity even though seed dehydration had started. This is because the increase of amylose content (mg per seed) of BEIIb-deficient mutant lines was more pronounced compared with that of the other lines after 30 DAF (Fig. 6D), whereas the amylopectin content (mg per seed) of these lines decreased after 20 DAF (Fig. 6E). These results suggest that there are two stages (before 20 DAF and from 30 DAF to maturity) during which amylose content rapidly increases in the *ss3a/be2b* mutant.

## Discussion

### Production of high-amylose starch in cereals

The amylose content definitely affects the physicochemical properties of starch (Singh *et al.*, 2006). High-amylose starches of maize are widely utilized for RS materials, low-calorie food manufacturing, and industrial applications such as a thickener, binder, and film (Li *et al.*, 2008; Jiang *et al.*, 2010*a*). Maize mutants deficient in BEIIb (Baba and Arai, 1984; Jane *et al.*, 1999), SSIIa (Perera *et al.*, 2001), and SSIII (Inouchi *et al.*, 1991) accumulate high-amylose starch in the endosperm. The BEIIb-deficient maize mutant *amylose extender* has 50–70% of the amylose content incorporated into endosperm starch. A deficiency of BEI and BEIIb results in the accumulation of >85% of amylose in maize endosperm starch (Li *et al.*, 2008). In wheat, RNA interference (RNAi) knockdown of *BEIIa* and *BEIIb* resulted in the accumulation of >70% of amylose in endosperm starch; these high-amylose starches improve the health of the large intestine of rats (Regina *et al.*, 2006). Using a chimeric RNAi hairpin of the branching isozyme genes *BEI*, *BEIIa*, and *BEIIb* resulted in the production of amylose-only starch granules in barley endosperm (Carciofi *et al.*, 2012). The regulation of *BEI* and *BEIIb* in rice by RNAi technology also resulted in the production of >60% amylose-starch (estimated by a colorimetric iodometric method), and these high-amylose starches were enriched for RS (Zhu *et al.*, 2012). Transgenic rice plants that introduced the *Wx* ^*a*^ gene (the wild type of the *GBSSI* gene) into the *ss2a*/*ss3a* line having the *Wx* ^*b*^ gene (the leaky mutant of the *GBSSI* gene), and that reduced *BEIIb* expression by artificial micro RNA, had >40% of the amylose content in endosperm starch (Butardo *et al.*, 2011; Crofts *et al.*, 2012). Although reports of high-amylose starch in rice are limited to genetically modified (GM) plants, the highest AACs in starches of non-GM rice cultivars were ~33% (Inouchi *et al.*, 2005). The high-amylose rice mutants generated from SSIIa-inactive japonica rice in the authors’ laboratory contained 30% amylose for *ss3a* (Fujita *et al.*, 2007), 28% for *be2b* (Abe *et al.*, 2014), and 33% for *ss1* ^*L*^ */ss3a* (Fujita *et al.*, 2011). These previous reports implied that the deficiencies of BE isozymes lead to the reduction of amylopectin synthesis and the deficiencies of SS isozymes lead to the enhancement of GBSSI. These effects should result in the high amylose content in the storage starches.

The AAC of *ss3a/be2b* (45%, estimated by a gel filtration method) isolated in this study is the highest in the non-GM mutant lines of rice so far reported. The long chains with DP >24 connecting the clusters of amylopectin in *ss3a/be2b* were more abundant compared with those in the wild type, but less abundant than those in *be2b* (Fig. 4B). Therefore, it is possible that the distinct starch structure and components of *ss3a/be2b* could be utilized for the production of low-calorie foods and for industrial applications such as biodegradable plastic.

**Fig. 7.**
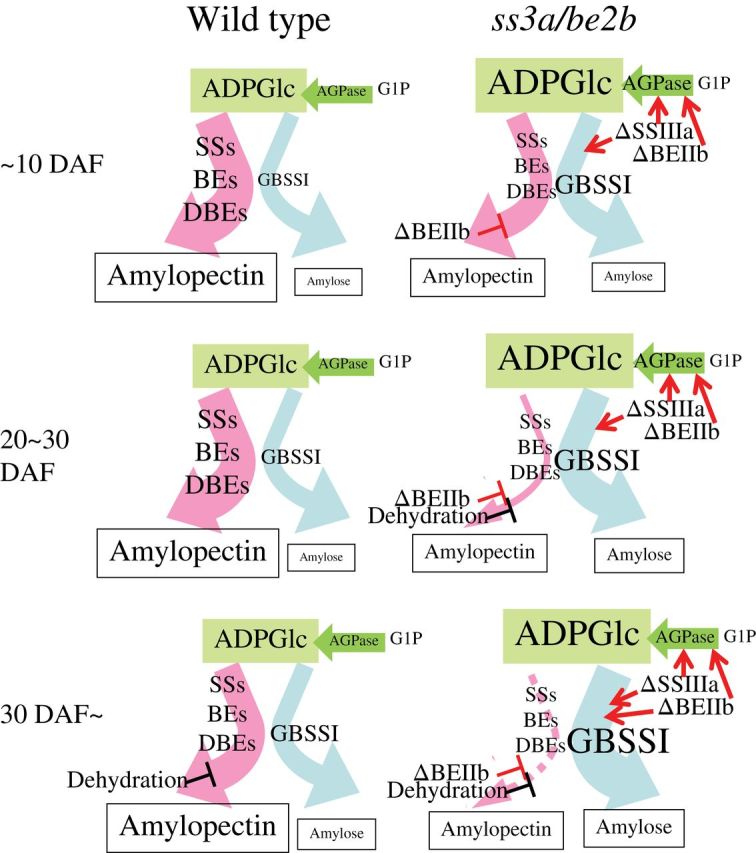
Possible mechanism of the partitioning of carbon into amylopectin and amylose during starch biosynthesis in the wild type and the *ss3a/be2b* mutant rice line. The sizes of the characters correspond to the expression levels of enzymes and the amount of ADP-glucose. The thickness of the arrows shows the amount of carbon flow into amylopectin and amylose. ΔSSIIIa and ΔBEIIb indicate the deficiency of SSIIIa and BEIIb, respectively. Red arrows and slanting ‘T’s indicate the enhancement and suppression of enzyme levels, respectively. It should be noted that the flow to the ADP-glucose and/or the level of ADP-glucose concentration are increased by the defect of SSIIIa and BEIIb. ADPase, ADP-glucose pyrophosphorylase; ADPG, ADP-glucose; G1P, glucose-1 phosphate; SSs, starch synthases; BEs, branching enzymes; DBEs, debranching enzymes.

There are three reasons for the high amylose content in *ss3a/be2b* (Fig. 7). (i) The increase in the amount of GBSSI protein in the TBP fraction of SSIIIa-deficient mutant lines at 12 DAF (Fig. 3, TBP) and the high GBSSI activity of SSIIIa-deficient lines at 10 DAF (Table 1) resulted in an amylose content that was significantly higher than that of the wild type at 10–20 DAF (Fig. 6C). (ii) The significantly higher AGPase activity resulting from SSIIIa and BEIIb deficiency (Table 1) should lead to a high concentration of ADP-glucose in the amyloplast; GBSSI has a higher *K*_m_ for ADP-glucose than the other soluble SS isozymes in potato (Clarke *et al.*, 1999). If this is the same in the case of rice, amylose synthesis would also be enhanced in rice endosperm. (iii) Amylopectin biosynthesis stops at 20 DAF when seed dehydration begins in BEIIb-deficient mutant lines (Fig. 6E), whereas amylose biosynthesis continues after 30 DAF (Fig. 6C, D). GBSSI protein levels of *be2b* and *ss3a/be2b* mature seeds were also significantly higher than those of the wild type (Fig. 3; Table 2). This result implies that the GBSSI activities keep on synthesizing amylose after 30 DAF in BEIIb-deficient mutant lines, although the GBSSI activities after 30 DAF were not measured in this study. These results suggest that amylose synthesis was significantly enhanced in the endosperm of *ss3a/be2b* compared with mutants and the wild type (Fig. 7).

All the rice lines used in this study express inactive SSIIa enzyme originating from japonica rice cultivars. Therefore, the effects of abolishing SSIIIa and BEIIb in the presence of active SSIIa remain unknown. In order to define the effect of either the presence or absence of SSIIa activity under *ss3a/be2b*, it will be required either to introduce the *SSIIa* gene into the *ss3a/be2b* mutant used in this study or to abolish SSIIIa and BEIIb in indica rice cultivars.

The excessive chain elongation and imbalance between SS and BE activities resulting from BEIIb deficiency may be counteracted by the reduced SSI activity in *ss1* ^*L*^ */be2b*, and the higher amylopectin biosynthesis in the mutant compared with that of the *be2b* mutant (Abe *et al.*, 2014). In contrast, the SSI activity in *ss3a/be2b* was higher than that in the *be2b* mutant (Fig. 2A), and higher amylopectin biosynthesis was not observed in *ss3a/be2b* (Fig. 6E). The larger grain weight in *ss3a/be2b* compared with that in *be2b* (Table 2) should result from amylose biosynthesis in the endosperm until 20 DAF and from 30 DAF to maturity (Fig. 6C, D). The barley *amo1/sex6* mutant line, which is thought to be the mutant of *SSIIIa*/*SSIIa* genes, showed a significant increase in starch content relative to the *sex6* mutant line (Li *et al.*, 2011). They suggested that SSIIIa is acting as a negative regulator of starch biosynthesis. Further studies on the relationships between isozyme-related starch biosynthesis using multiple mutant lines are necessary for maximum accumulation of starch and regulation of the amylose content.

### The effects of SSIIIa and/or BEIIb deficiency on enzyme binding to starch granules

Previous studies reported that the extent of SSI and BEI binding to starch granules changed in BEIIb-deficient mutant lines in maize (Liu *et al.*, 2009) and rice (Abe *et al.*, 2014). SSIII in maize is a component of high molecular weight protein complexes (Hennen-Bierwagen *et al.*, 2009). In this study of rice, it appeared that BEIIb deficiency resulted in the binding of SSIIIa to starch granules, whereas this was not observed in wild-type cultivars (Fig. 3). Liu *et al.* (2012) showed that in maize, at least SSI, SSIIa, and BEIIb form a protein complex in the stroma in a phosphorylation-dependent manner and that these protein–protein complexes are trapped in starch granules during starch biosynthesis. They also described that granule-bound BEI and BEIIb in maize endosperm were completely phosphorylated. They hypothesized that these complexes are the functional components involved in the synthesis of amylopectin clusters (Liu *et al.*, 2009). Therefore, a deficiency of rice SSIIIa in addition to SSI, BEI, and BEIIb, or having inactive SSIIa may have altered the binding of other starch biosynthetic isozymes to the starch granules in rice.

## Supplementary data

Supplementary data are available at *JXB* online.

Figure S1. Observations of starch granules and endosperm cells.

Table S1. The composition of carbohydrate content (weight %) in the endosperm starch fractions separated by gel filtration chromatography (Toyopearl HW55S/HW50S×3).

Supplementary Data

## Abbreviations:

AAC: apparent amylose content
AGPase: adenosine diphosphate (ADP)-glucose pyrophosphorylase
BE: branching enzyme
CBB: Coomassie brilliant blue
DAF: days after flowering
DP: degree of polymerization
ELC: extra-long chain
GBSSI: granule-bound starch synthase I
LBP: loosely bound protein
PAGE: polyacrylamide gel electrophoresis
RS: resistant starch
SDS: sodium dodecyl sulphate
SP: soluble protein
SS: starch synthase
TBP: tightly bound protein.
