# Supplementary material for: Deficiencies in both starch synthase IIIa and branching enzyme IIb lead to a significant increase in amylose in SSIIa-inactive japonica rice seeds
Source: J Exp Bot. 2014 Jul 28;65(18):5497–507. doi: 10.1093/jxb/eru310 (PMC4157723; doi:10.1093/jxb/eru310)
Supplement: Supplementary Data [file supp_65_18_5497__index.html]

Deficiencies in both starch synthase IIIa and branching enzyme IIb lead to a significant increase in amylose in SSIIa-inactive japonica rice seeds — Deficiencies in both starch synthase IIIa and branching enzyme IIb lead to a significant increase in amylose in SSIIa-inactive japonica rice seeds — Supplementary Data 

# Deficiencies in both starch synthase IIIa and branching enzyme IIb lead to a significant increase in amylose in SSIIa-inactive japonica rice seeds

## Supplementary Data

Data files

**Files in this Data Supplement:**

- Supplementary Data - Supplementary Data
