# Supplementary material for: Deficiencies in both starch synthase IIIa and branching enzyme IIb lead to a significant increase in amylose in SSIIa-inactive japonica rice seeds
Source: J Exp Bot. 2014 Jul 28;65(18):5497–507. doi: 10.1093/jxb/eru310 (PMC4157723; doi:10.1093/jxb/eru310)
Supplement: Supplementary Data [file supp_eru310_jexbot123794_file001.pdf]

Deficiencies in both starch synthase (SS) IIIa and branching enzyme IIb lead to a significant increase in amylose in SSIIa inactive japonica rice seeds.

Hiroki Asai, Natsuko Abe, Ryo Matsushima, Naoko F Oitome, Naoko Crofts, Yasunori Nakamura, Naoko Fujita

SUPPLEMENTARY DATA

Supplementary Table 1. The composition of carbohydrate content (weight %) in the endosperm starch fractions separated by gel filtration chromatography (Toyopearl HW55S/HW50S x 3).

Lines		Frac. I[a] (%)	Frac. II (%)	Frac. III (%)	TAC[e] (%)	III/II
WT	starch[b]	21.2±0.3[d,h]	20.3±0.1[h]	58.5±0.3[h]	18.2[h]	2.9±0.0[h]
(Nipponbare)	amylopectin[c]	3.0±0.4	17.7±0.9[h]	54.4±1.8[h]	-	3.1±0.1[h]
WT	starch	21.6±2.0[h]	21.0±0.7[h]	57.4±2.0[h]	19.3[h]	2.8±0.2[h]
(Kinmaze)	amylopectin	2.3±0.3	21.3±0.7[h]	59.7±2.3[h]	-	2.8±0.2[h]
ss3a	starch	30.7±1.0[f,g]	13.9±0.9[f,g]	55.4±0.9[f,g]	27.6[f,g]	4.0±0.3[f,g]
(*e1*)	amylopectin	3.1±0.3	12.1±0.5[f,g]	53.1±1.3[g]	-	4.4±0.1[f,g]
be2b	starch	28.1±0.1[g]	39.7±0.2[f,g]	32.2±0.3[f,g]	26.8[g]	0.8±0.0[f,g]
(*EM10*)	amylopectin	1.3±0.0[f,g]	38.3±0.4[f,g]	32.3±0.4[f,g]	-	0.8±0.0[f]
ss3a/be2b	starch	45.1±1.5	27.5±0.3	27.4±1.1	42.6	1.0±0.0
(*#4019*)	amylopectin	2.5±0.1	27.2±0.4	24.4±0.3	-	0.9±0.0

[a]Three fractions (Fr. I, II and III) was divided at the valleys of the carbohydrate content curve equipped with refractive index detectors (Figure 5).

[b]Total carbohydrate content was 100 %.

[c]The area for Fr. II and Fr. III of amylopectin were superimposed on those of the starch, and the amount of the Fr. I of amylopectin (extra long chain) was calculated.

[d]Mean±SE of at least two replications.

[e]True amylose content=apparent amylose content (Fr. I of starch) – extra long chains (Fr. I of amylopectin).

[f]Significant differences between parental mutant lines and the WT by t-test at $P<0.05$.

[g]Significant differences between the parental mutant lines and the *ss3a/be2b* mutant by t-test at $P<0.05$.

[h]Significant differences between the *ss3a/be2b* mutant line and the WT by t-test at $P<0.05$.

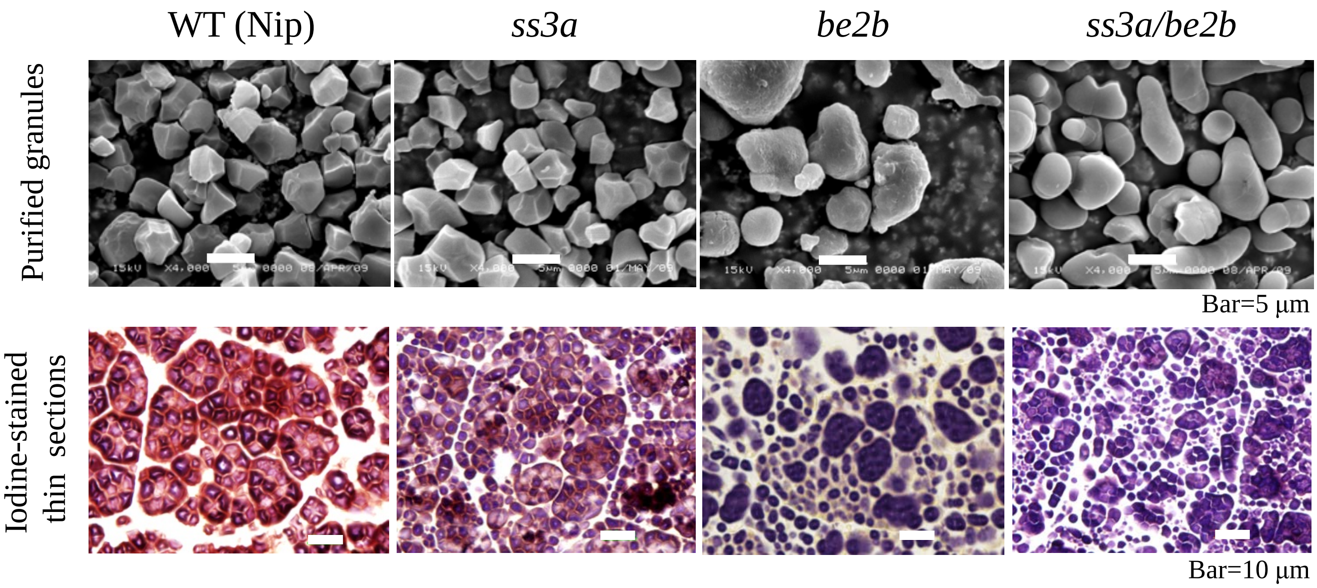

Supplementary Figure 1. Observations of starch granules and endosperm cells. Scanning electron micrographs of purified starch granules (upper panels). Bars=5 µm. Thin-section of mature endosperm stained with iodine observed by light microscopy (lower panels). Bars=10 µm.
